# The Association Between Suicide Attempts, Anxiety, and Childhood Maltreatment Among Adolescents and Young Adults With First Depressive Episodes

**DOI:** 10.3389/fpsyt.2021.745470

**Published:** 2021-12-17

**Authors:** Hui Chen, Wen Li, Xia Cao, Peiqu Liu, Jiali Liu, Xianliang Chen, Chenyuli Luo, Xiaoxi Liang, Huijuan Guo, Shaoling Zhong, Xiaoping Wang, Jiansong Zhou

**Affiliations:** ^1^National Clinical Research Center for Mental Disorders, Department of Psychiatry, The Second Xiangya Hospital of Central South University, Changsha, China; ^2^Shanghai Key Laboratory of Forensic Medicine, Key Laboratory of Forensic Science, Ministry of Justice China, Shanghai Forensic Service Platform, Academy of Forensic Science, Shanghai, China; ^3^Health Management Center, Health Management Research Center of Central South University, The Third Xiangya Hospital, Central South University, Changsha, China; ^4^Dongguan Mental Health Center, Dongguan, China

**Keywords:** suicide attempt, first depressive episode, anxiety, adolescents, childhood maltreatment

## Abstract

**Objective:** Adolescents and young adults are susceptible to high-risk behaviors such as self-harm and suicide. However, the impact of childhood maltreatment on suicide attempts in adolescents and young adults with first episode of depression remains unclear. This study examined the association between suicide attempts and childhood maltreatment among adolescents and young adults with first depressive episodes.

**Methods:** A total of 181 adolescents and young adults with first depressive episodes were included. The Child Trauma Questionnaire (CTQ), Beck Anxiety Inventory (BAI), and Patient Health Questionnaire-2 (PHQ-2) were used to assess childhood maltreatment and the severity of anxiety and depressive symptoms, respectively. The suicide item in the MINI-International Neuropsychiatric Interview (M.I.N.I.) 5.0 was used to assess the suicide attempts. Logistic regression analyses were used to explore the associated factors of suicide attempts.

**Results:** The prevalence of SA in the total sample was 31.5% (95% CI = 24.9–38.1%). Multivariate logistic regression analyses revealed that the diagnosis of bipolar disorder (OR = 2.18, 95% CI = 1.07–4.40), smoking (OR = 2.64, 95% CI = 1.10–6.37), anxiety symptoms (OR = 1.05, 95% CI = 1.02–1.08), and childhood maltreatment (OR = 1.04, 95% CI = 1.01–1.07) were potential associated factors of SA. In addition, anxiety symptoms had a mediating effect on the relationship between childhood maltreatment and SA.

**Conclusion:** Adolescents and young adults with first depressive episodes and having experiences of childhood maltreatment are at a high risk of suicide. The severity of anxiety symptoms may mediate the relation between childhood maltreatment and suicide attempts in this group of patients.

## Introduction

Suicide attempt (SA), one of the major suicidal behaviors, is defined as an act of self-injuring with the intention of ending one's own life (1). Around 703,000 people die from suicide worldwide, each year. Suicide is a leading cause of death among young people aged 15–24 years and suicide attempts are considerably high among children and adolescents aged 12–17 years (2, 3). The prevalence of SA among Chinese adolescents ranges from 0.94 to 9.01%, with an overall prevalence of 2.94% (95% CI = 2.53–3.41%) (4).

Mental disorders, such as major depressive disorder (MDD) and bipolar disorder (BP), could increase the risk of suicide behaviors in adolescents and young adults (5–7). A meta-analysis found that MDD and BP were the most common diagnoses among youths with SA (8, 9). The risk of SA is five times higher in adolescents with MDD than in the general population (10). The lifetime prevalence of SA in younger BP patients (14.7%, 95% CI = 5.9–20.0%) (11) was significantly higher than those without psychiatric disorders (0.8%, 95% CI = 0.7–0.9%) (12). For patients with BP, suicide behaviors were predominantly present during depressive episodes (13), especially during the first depressive episode (14).

Apart from the negative outcomes of physical injury and the increased risk of completed suicide, SA could worsen patients' general well-being and increase their utilization of health services and the economic burden (15). Understanding the associates of SA could benefit the development of effective prevention strategies for adolescents and young adults with MDD and BP.

Childhood maltreatment, including abuse (i.e., physical, sexual, and emotional abuse) and neglect (i.e., physical and emotional neglect), often has negative and long-term adverse effects on individuals' mental health (16–18). Increasing evidence shows that childhood maltreatment is closely related to the following self-harm and suicide behaviors including suicide attempts. Such phenomenon has also been confirmed among patients with MDD and BP (19, 20). For example, childhood maltreatment, such as physical abuse and emotional neglect, could increase the risk of subsequent SA among patients with MDD and BP (21, 22). Several theories have been raised to explain the path from childhood maltreatment to SA. For instance, childhood maltreatment can disturb developmental processes associated with strengthening of emotional regulation and relevant interpersonal skills and increased impulsivity and neuroticism. These disturbances may lower the threshold of suicidal behavior in individuals to experience stressful events. And the reduction in long-term social support associated with childhood maltreatment also increases the risk of suicide attempts (23). Previous studies majorly examined the association between SA and childhood maltreatment among adults with MDD and BP (22, 24). However, limited studies have discussed this relationship among adolescents and young adults. Besides, the existing studies are majorly conducted in Western populations, which may limit their generalization among other racial groups including China (20).

Considering that childhood maltreatment may lead to SA among patients with MDD and BP and suicide behaviors are most likely presented in their first depressive episode (14, 25), we conducted this study to examine the relationship between childhood maltreatment and SA among adolescents and young adults with first depressive episodes. Antidepressants, such as buprenorphine (26) and ketamine (27) and cognitive behavioral therapy (CBT) can reduce self-harming behaviors in depressed patients (28, 29). For antidepressants, which can increase 5-HT neurotransmission and 5-HT1A autoreceptor sensitivity, release hopelessness feelings and reduce depression, achieve the effect of reducing suicide-related behavior (30). For CBT, aims to change feelings of negativity and behaviors into positive thoughts and behaviors to redress adverse cognitive and decrease negative emotional affects (31). Therefore, we targeted on psychiatric treatment -naive patients first depressive episode in this study to avoid the impact of psychiatric medication and psychotherapy on suicide behaviors. For both patients and clinical practitioners, this study will try to verify the understanding on the impact of childhood maltreatment experience by using a purer sample and strengthen the evidence of intervening childhood maltreatment at an early age.

In addition, studies among healthy adults suggested that negative emotion, such as depression and anxiety, may mediate the association between childhood maltreatment and suicide behaviors (32–34). Such association is still under discussed among patients with depression. Therefore, we further explored the mediation effect of depressive and anxiety symptoms between childhood maltreatment and SA in our studied population.

## Materials and Methods

### Participants

This cross-sectional study is part of Youth Depression Cohort (XiangyYa) study (YDC-XY), which was conducted from January 1, 2018, to December 31, 2019, at the outpatient department of the Second Xiangya Hospital of Central South University, China. Patients who visited the hospital because of their first depressive episode were consecutively recruited if they fulfilled the following criteria: (1) aged between 14 and 24, (2) experiencing their first depressive episode, (3) diagnosed with MDD or BP according to the Diagnostic and Statistical Manual of Mental Disorders-V (DSM-V), and (4) received no psychiatric treatment (including antipsychotic drugs and psychotherapy) in the past 3 months. The exclusion criteria were as follows: (1) comorbid with neurological conditions or substance dependence and (2) participating in other clinical trials.

### Measurements

Sociodemographic and clinical data were collected, including age, gender, education year, marital status, nationality, occupation, history of smoking, drinking, drug use, family history of psychiatric disorders and parents' marital status. Drug use history was assessed using a dichotomous item: “In your lifetime, have you ever used any of the following drugs (according to the MINI-International Neuropsychiatric Interview, M.I.N.I)?.” Smoking and drinking history was assessed using two dichotomous items: “Have you ever smoked?” and “Have you ever consumed alcohol?.” SA was assessed using the item of M.I.N. I: “In the past month did you attempt suicide?.”

The Child Trauma Questionnaire (CTQ) (35), a 28-item self-report instrument, was used to assess the experience of abuse and neglect in childhood and adolescence. The scale was first developed by Bernstein in 1998 and has been widely used. Its Chinese version has been validated in both adolescents and adults (36). The CTQ assesses childhood maltreatment from five aspects, including emotional neglect, emotional abuse, physical neglect, physical abuse, and sexual abuse. Each subscale consists of five five-point items, with each item rated from “1” (not at all) to “5” (very often). The scores of each subscale were the summation of all loaded item scores and ranged from 5 to 25, while the total score of the CTQ was the summation of all subscale scores and ranged from 25 to 125.

The Beck Anxiety Inventory (BAI) (37) was used to assess the anxiety symptoms. It is a self-rating scale consisting of 21 four-point items with each item rated from “0” (not at all) to “3” (severely), and the total score of the scale ranged from 0 to 63, with a higher total score indicating more severe anxiety symptoms. The Chinese version of the BAI was found to have satisfactory reliability and validity in adolescents and adults (38).

The Chinese version of the Patient Health Questionnaire-9 has been widely used in research and clinical practice to screen depressive symptoms (39). The Patient Health Questionnaire-2 (PHQ-2) (40) is a short version of the PHQ-9 and has been found to have equal psychometric properties compared to the PHQ-9 for depressive symptom screening (41). The two items of the PHQ-2 were rated from “0” (none at all) to “3” (almost every day), and the total score of the scale ranged from 0 to 6.

### Procedures

The continuous sampling method was applied. All patients who were first time visit the psychiatric outpatient services because of depression invited to participate in the survey. After obtaining written informed consent from the participants, a face-to-face interview was conducted to reconfirm the psychiatric diagnosis by a senior psychiatrist using a structured diagnostic tool, the M.I.N.I according to the DSM-V. Then all participants were asked complete all measurements mentioned above. According to patients' response to the item “In the past month did you attempt suicide?” in M.I.N.I, patients were recognized as having “suicide attempts” if they answer “yes” and as having “no suicide attempts” if “no.”

### Statistical Analysis

The SPSS 24.0 software was used for all statistical analyses. The two-independent sample *t*-tests and chi-square tests were used to compare the socio-demographic and clinical data between patients with and without SA, as appropriate. Multiple logistic regressions were performed to examine the independent variables associated with SA. Variables with *p* < 0.10 in univariate analyses were entered into the regression model as independent variables. In addition, the association between the total score of the BAI and CTQ was tested using Pearson's correlation coefficient. Mediation analysis was conducted using the PROCESS v3. 3 to examine the extent to which potential mediators may explain the relationship between childhood maltreatment and SA. Potential mediators were selected based on the results of the multiple logistic regression. The significance level was set as *p* < 0.05 (two sides).

### Ethical Approval and Informed Consent

This study was approved by the ethics committee of The Second Xiangya Hospital of Central South University. All patients signed written informed consent.

## Results

A total of 181 adolescents and young adults with first depressive episodes met the eligibility criteria and were included. Among them, 31.5% (95% CI = 24.9–38.1%) (*n* = 57) reported SA. The sociodemographic and clinical data are shown in Table 1. The majority of the patients were female (75.1%). The mean age of the patients was 18.6 ± 2.3 years, while the mean years of education was 12.2 ± 2.3 years. A total of 115 (63.5%) patients were diagnosed with MDD, while the remaining 66 (36.5%) patients were diagnosed with BP.

**Table 1:** Sociodemographic and clinical characteristics among adolescents and young adults with first depressive episode.

**Variables**	**Total sample**		**Non-SA**		**SA**		**Statistics**	
	**Mean**	**SD**	**Mean**	**SD**	**Mean**	**SD**	***t***	***P***
Age (year)	18.6	2.3	18.9	2.2	18.2	2.4	0.134	0.055
Education year	12.2	2.3	12.2	2.3	12.1	2.3	0.010	0.661
The PHQ-2 total score	3.2	1.1	3.1	1.1	3.3	1.2	1.840	0.405
The BAI total score	25.8	11.5	23.5	10.9	30.9	11.0	0.023	<0.001
The CTQ total score	51.2	13.6	48.5	12.1	57.1	14.8	3.677	<0.001
**The CTQ subscale score**								
Emotional abuse	10.9	4.4	10.3	4.0	12.7	4.7	1.668	<0.001
Physical abuse	7.2	3.5	6.9	3.2	8.1	3.9	5.017	0.033
Sexual abuse	6.0	2.1	5.9	2.0	6.4	2.3	2.585	0.118
Emotional neglect	16.9	5.3	16.2	5.0	18.4	5.7	1.733	0.007
Physical neglect	10.1	3.3	9.5	3.0	11.5	3.8	3.656	<0.001
	***N***	**%**	**N**	**%**	**N**	**%**	***X*** **^2^**	***P***
Diagnosis							9.387	0.002
MDD	115	63.5	88	71.0	27	47.4		
BP	66	36.5	36	29.0	30	52.6		
**Gender**								
Male	45	24.9	34	27.4	11	19.3	1.379	0.240
Female	136	75.1	90	72.6	46	80.7		
**Nationality**								
Ethnic Han	167	92.7	113	91.1	54	94.7	0.712	0.399
Others	14	7.7	11	8.9	3	5.3		
Occupation							0.137	0.711
Students	155	85.6	107	86.3	48	84.2		
Others	26	14.4	17	13.7	9	15.8		
Drinking							4.342	0.037
No	141	77.9	102	82.3	39	68.4		
Yes	40	22.1	17	17.7	18	31.6		
Smoking							4.264	0.039
No	149	82.3	107	86.3	42	73.7		
Yes	32	17.7	17	13.7	15	26.3		
Family history							1.264	0.261
No	154	85.1	103	83.1	51	89.5		
Yes	27	14.9	21	16.9	6	10.5		
Marital relationship of parents				2.911	0.406			
Good	103	56.9	72	58.1	31	54.4		
Bad	37	20.4	28	14.5	9	15.8		
Divorced	32	17.7	19	15.3	13	22.8		
Widow	9	5.0	5	4.0	4	7.0		

*SA, suicide attempt; SD, standard deviation; BP, bipolar disorder; MDD, major depressive disorder; BAI, Beck Anxiety Inventory; CTQ, Child Trauma Questionnaire; PHQ-2, Patient Health Questionnaire-2*.

Patients with SA were more likely to have a diagnosis of BP (52.6 vs. 29.0%, *p* = 0.002), drink (31.6 vs. 17.7%, *p* < 0.050), smoke (26.3 vs. 13.7%, *p* < 0.050), and present with more severe anxiety symptoms (30.9 ± 11.0 vs. 23.5 ± 10.9, *p* < 0.001) than those without. Compared to patients without SA, patients with SA experienced significantly more severe emotional abuse (12.7 ± 4.7 vs. 10.3 ± 4.0, *p* < 0.001), physical abuse (8.1 ± 3.9 vs. 6.9 ± 3.2, *p* = 0.033), emotional neglect (18.4 ± 5.7 vs. 16.2 ± 5.0, *p* < 0.001), physical neglect (11.5 ± 3.8 vs. 9.5 ± 3.0, *p* < 0.001), and overall childhood maltreatment (57.1 ± 14.8 vs. 48.5 ± 12.1, *p* < 0.001). There was no significant difference in the PHQ-2 total score between patients with and without SA (3.3 ± 1.2 vs. 3.1 ± 1.1, *p* = 0.405) (Table 1).

A multiple logistic regression model revealed that the diagnosis of BP [odds ratio (OR) =2.18, 95% CI = 1.07–4.40], smoking (OR = 2.63, 95% CI = 1.10–6.37), severity of anxiety symptoms (OR = 1.05, 95% CI = 1.02–1.08), and childhood maltreatment (OR = 1.04, 95% CI = 1.01–1.07) were significantly and positively associated with SA (Table 2). There was a significant positive association between the severity of anxiety symptoms and overall childhood maltreatment (*r* = 0.201, *p* < 0.001).

**Table 2:** Multiple logistic regression model assessing variables associated with SAs among adolescents and young adults with first depressive episode.

**Variables**	***P***	**Odds ratio (OR)**	**95% Confidence interval** **(95% CI)**	
**Psychiatric diagnosis**				
MDD	Ref			
BP	0.031	2.175	1.074	4.404
Smoking	0.005	2.644	1.098	6.365
Drinking	0.320	1.691	0.600	4.764
BAI total score	0.005	1.049	1.015	1.084
CTQ total score	0.006	1.039	1.011	1.067

*SA, suicide attempt; BP, bipolar disorder; MDD, major depressive disorder; BAI, Beck Anxiety Inventory; CTQ, Child Trauma Questionnaire; Ref, reference group*.

**Figure 1:**
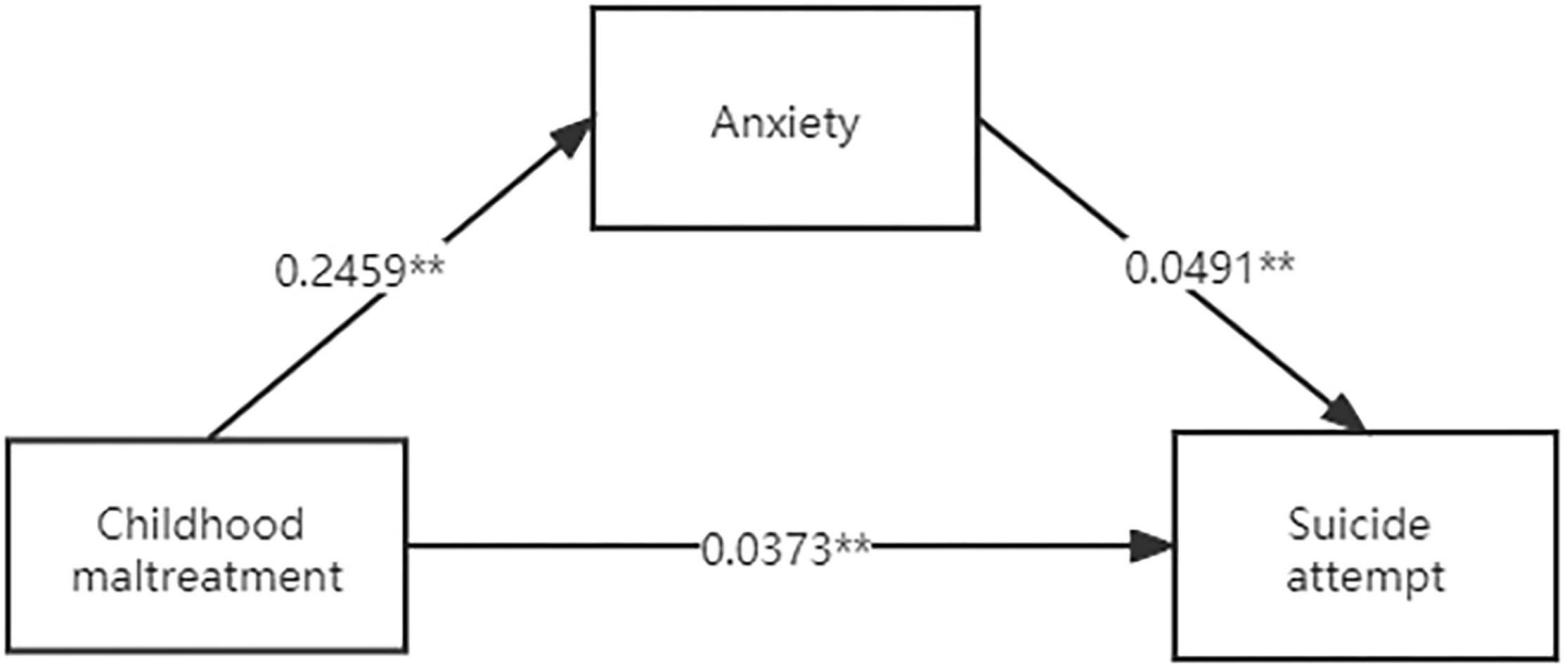
The mediation effect of anxiety on the relationship between childhood maltreatment and SA. **P < 0.01.

The mediation analysis revealed that anxiety symptoms mediated the relationship between childhood maltreatment and SA [coefficient (indirect) =0.01, standard error (SE) =0.005, 95% CI = 0.004–0.023] and explained 24.40% of the total effect (Figure 1, Table 3).

**Table 3:** The mediation effect of anxiety symptoms between childhood maltreatment and SA.

	**Effect**	**Boot SE**	**Boot LLCI**	**Boot ULCI**	**Proportion** **mediated %**
Direct effect	0.0373	0.0132	0.0114	0.0632	75.60
Indirect effect	0.0121	0.0050	0.0040	0.0231	24.40
Total effect	0.0494	0.0025	0.0052	0.0148	100

*SA, suicide attempt; LLCI, lower limit confidence interval; ULCI, upper limit confidence interval; SE, standard error*.

## Discussion

This study found that 31.5% of treatment-naïve adolescents and young adults with first depressive episodes had SA, which was higher than one previous study about the first-episode and drug naïve patients with MDD with a prevalence of 19.9% (42). Several possible reasons may contribute to this discrepancy. On the one hand, the patients in this study were untreated adolescents and young adults in the first episode of depression and acute episode, those who had severe depressive symptoms. On the other hand, our study recruited clinical psychiatric outpatients, those who had more severe depressive symptoms than those surveyed in the community. Adolescents and young adults are the most common age groups for first mood disorders (43, 44), such as MDD and BP. These people are not mature in terms of cognitive structure, emotional structure and rationality. When an adverse stressful event or major environmental change cannot be dealt with, it is possible they may decide to commit suicide to end the current pain and escape the existing situation (45).

One of the important findings of this study is that patients with SA experienced more childhood maltreatment than those without SA. Previous studies also found a similar association between childhood maltreatment and SA among general adolescents and adults (21, 46). Patients experiencing childhood maltreatment often have negative family environments, such as a lack of shelter and safe living conditions, which can prevent the patients from developing appropriate emotional regulation and stress coping skills (47). As a result, they may impulsively react to stressful life events and even present suicidal behaviors. Emotional trauma may negatively affect the development of the hypothalamus-pituitary-adrenal axis (HPA axis) in growth and progression, and alteration of the function of the HPA axis was found to have an impact on individuals' biological, emotional, behavioral, and cognitive responses to stressful events (48–52), which were related to suicidal behaviors.

Another important finding of this study is that anxiety symptoms were found to mediate the relationship between childhood maltreatment and SA among adolescents and young adults with first depressive episodes, which is consistent with previous studies (53). These results have been confirmed in previous studies (54–56), which found an independent association between SA and anxiety symptoms in adolescents. Anxiety can also predict the occurrence of SA (57). Behavioral avoidance, one clinical feature of anxiety, often leads to social isolation, reduced quality of life, and impaired functioning, all of which are related to an increased risk of suicide (58, 59). Relevant studies have shown that the influence of childhood maltreatment on SA is indirectly affected by psychological factors (34). This suggests that anxiety should be the treatment target in the prevention strategies of SA in depressed adolescents and young adults.

Furthermore, this study found that young patients with BP were more likely to have SA than those with MDD, which is consistent with previous findings (8, 60). For example, in a study of people hospitalized after SA, 28 percent were diagnosed with MDD and 39 percent with BP (61). In an 18-month follow-up cohort of patients with BP and MDD, 19.9% of those with BP and 9.5% of those with MDD attempted suicide (62). The possible reasons for the relatively higher risk of SA in patients with BP compared to their counterparts with MDD may be that they have more severe depressive symptoms during their depressive episodes (60), more difficulties of treatment, more impulsivity, and a higher risk of comorbidity of anxiety (63) than patients with MDD.

The strengths of the present study include the large sample size of treatment-naïve adolescents and young adults with first depressive episodes. In addition, the study examined the association between SA and childhood maltreatment among adolescents and young adults with MDD and BP, and discussed the association between childhood maltreatment and suicide behaviors among patients with depression. However, several methodological limitations should be considered. First, as a cross-sectional study, a causal relationship between SA and associated factors could not be generated. Second, some potential factors associated with SA, such as residential type and socioeconomic state (15, 64), were not examined. Third, the single-site study design could limit the generalization of the results. Forth, young patients with MDD may convert to BP during the progression of the disease, which may bias the estimation of the prevalence of SA in patients with each disorder. Fifth, although PHQ-2 has been proved to have equal psychometric properties compared to the PHQ-9 for depressive symptom screening, only using 2 items for depressive symptom screening may influence the identification of other depressive symptoms and the results of this study. Sixth, although we studied a pure sample that was treatment-naïve and with a first depressive episode, the acute illness phase may increase the risk of suicide behaviors in this population, which may potentially bias the results. Finally, some potential associated factors, such as smoking and drinking, were not included in the mediation model.

## Conclusion

This study found that treatment-naive adolescents and young adults with first depressive episodes were at a high risk of SA. Patients with SA were more likely to have experiences of childhood maltreatment and present more severe anxiety symptoms than those without SA. Anxiety symptoms may mediate the relationship between childhood maltreatment and SAs. Considering the severe negative outcomes of SA, it is essential to develop prevention strategies for SA among depressed adolescents and young adults. Alleviating anxiety may be an effective way to reduce the risk of suicide in this group of patients.

## Data Availability Statement

The original contributions presented in the study are included in the article/supplementary material, further inquiries can be directed to the corresponding authors.

## Ethics Statement

The studies involving human participants were reviewed and approved by the Ethics Committee of the Second Xiangya Hospital of Central South University. Written informed consent to participate in this study was provided by the participants' legal guardian/next of kin.

## Author Contributions

HC, SZ, and JZ: conceived and designed the study. PL, JL, XCh, HG, CL, and XL: participated in the acquisition of data. HC: analyzed the data and drafted the manuscript. WL, XCa, JZ, and XW: revised the manuscript. All authors read and approved the final manuscript.

## Funding

This study was supported in part by grants from the National Natural Science Foundation of China (82071543, JZ, PI), the Natural Science Foundation of Hunan (2019JJ40424, Jiansong Zhou, PI), the Health Committee of Hunan (202103091470, JZ, PI), the Hunan Province Innovation Province Construction Project (2019SK2334, XW and JZ, Co-PI), and Clinical medical technology innovation guidance project of Hunan (2020SK53415, JZ, PI).

## Conflict of Interest

The authors declare that the research was conducted in the absence of any commercial or financial relationships that could be construed as a potential conflict of interest.

## Publisher's Note

All claims expressed in this article are solely those of the authors and do not necessarily represent those of their affiliated organizations, or those of the publisher, the editors and the reviewers. Any product that may be evaluated in this article, or claim that may be made by its manufacturer, is not guaranteed or endorsed by the publisher.
